# Single nucleotide polymorphism (SNP) analysis used for the phylogeny of the *Mycobacterium tuberculosis* complex based on a pyrosequencing assay

**DOI:** 10.1186/1471-2180-14-21

**Published:** 2014-02-03

**Authors:** Adriana Cabal, Mark Strunk, José Domínguez, María Antonia Lezcano, María Asunción Vitoria, Miguel Ferrero, Carlos Martín, María José Iglesias, Sofía Samper

**Affiliations:** 1 IIS Aragón, Hopsital Universitario Miguel Servet, Zaragoza, Spain; 2 IIS Aragón, CIBER de Enfermedades Hepáticas y Digestivas, Zaragoza, Spain; 3 Institut d’Investigació Germans Trias i Pujol, Badalona, Spain; 4 Universitat Autònoma de Barcelona, Barcelona, Spain; 5 CIBER de Enfermedades Respiratorias, Madrid, Spain; 6 Hospital Universitario Lozano Blesa, CIBER de Enfermedades Respiratorias, Zaragoza, Spain; 7 Hospital San Jorge, Huesca, Spain; 8 Universidad de Zaragoza, CIBER de Enfermedades Respiratorias, Zaragoza, Spain; 9 Instituto Aragonés de Ciencias de la Salud, Zaragoza, Spain; 10 Hospital Miguel Servet – IIS Aragón, Laboratorio de Investigación Molecular, P. Isabel la Catolica 1-3, Zaragoza 50009, Spain

**Keywords:** *M. tuberculosis*, SNP, Pyrosequencing, SCG, Lineages, Cluster

## Abstract

**Background:**

Different polymorphisms have been described as markers to classify the lineages of the *Mycobacterium tuberculosis* complex. The analysis of nine single nucleotide polymorphisms (SNPs) was used to describe seven SNPs cluster groups (SCGs). We attempted to classify those strains that could not been categorized into lineages by the genotyping methods used in the routine testing.

**Results:**

The *M. tuberculosis* complex isolates collected in 2010 in our region were analysed. A new method based on multiplex-PCRs and pyrosequencing to analyse these SNPs was designed. For the pyrosequencing assay nine SNPs that defined the seven SCGs were selected from the literature: 1977, 74092, 105139, 232574, 311613, 913274, 2460626, 3352929 *and gyrA*95. In addition, SNPs in *kat*G^463^, *mgtC*^*182*^, *Ag85C*^103^ and RD^Rio^ deletion were detected.

**Conclusions:**

This work has permitted to achieve a better classification of Aragonian strains into SCGs and in some cases, to assign strains to its certain lineage. Besides, the description of a new pattern shared by two isolates “SCG-6c” reinforces the interest of SNPs to follow the evolution of *M. tuberculosis* complex.

## Background

The species of the *Mycobacterium tuberculosis* complex (MTC) show a 99.9% of similarity in their nucleotide sequence and their *16SrRNA* do not differ between members, only *M. canetti* does [1]. Despite this identity in their genomes, a large number of long sequence polymorphisms (LSPs), a variation in repetitive elements in the genome, and single nucleotide polymorphisms (SNPs) have been detected [2,3]. It is the diversity of such polymorphisms, which is taken for phylogenetic studies with clinical isolates. In 1997, Sreevatsan et al. based on the presence of two SNPs in *gyrA*^95(AGC→ACC)^ and *katG*^463(CGC→CTG)^, classified all MTC isolates into three principal genetic groups or PGGs [4]. Afterwards, Brudey et al. based on the “Direct Repeat” locus (DR) diversity detected by Spoligotyping, classified thousands of MTC clinical strains isolated worldwide in different lineages or families [5]. These families were named according with their main geographical origin; Latin American-Mediterranean family (LAM) isolates, which are the cause of 15% of the new TB (tuberculosis) cases detected each year worldwide, are highly prevalent in Latin America and the Mediterranean area [6,7]. Within this family a sub-lineage has been characterized by a genomic deletion known as RD^Rio^, which was firstly detected in Brazil, but it was widely spread throughout the world [8,9]. Haarlem family is ubiquitous throughout the world and accounts for 25% of the isolates extracted in Europe, Central America and the Caribbean [10]. The T family is an “ill defined” family that was characterized by default. It includes over 600 shared international types (SITs) and it has been divided into 5 subgroups, from T1 to T5 [5,7]. Beijing family has become significant due to several multidrug-resistant (MDR) outbreaks identified [11]. S family was identified predominantly in patients of Italian origin [7]. “X” family was described to be highly prevalent in North America (21.5%) and Central America (11.9%), although some researchers correlate it with African-Americans [5]. Central Asian family (CAS) has been identified mostly in India, where presents a common sub-lineage called CAS-1 [7]. East African Indonesian family (EAI) has a higher prevalence in Southeast Asia, particularly in The Philippines, Malaysia, Vietnam and Thailand [12,13]. Finally, the U family (Undefined) does not meet the criteria of the other described families and it is considered separately [5]. Furthermore, a set of SNPs has been published as markers with phylogenetic value. Thus, seven phylogenetically different SNP cluster groups (SCGs) with 5 subgroups have been defined based on a set of SNPs, which have been related to the previously defined families [14-16]. Other significant polymorphisms were described as markers for particular families. By way of illustration, SNP in *Ag85C*^103(GAG→GAA)^ has been associated with LAM family strains [8] and among these strains a genomic deletion known as RD^Rio^ has been defined [9]. Likewise, some specific polymorphisms in *ogt*^*44(ACC*→AGC*)*^, ung501^*501(CTG*→*CTA)*^ and *mgtC*^*182(CGC*→*CAC)*^ could serve as genetic markers for Haarlem family [17,18]. Finally, a global phylogeny for *M. tuberculosis* was described based on LSPs by six phylogeographical lineages, besides the *M. bovis* and *M. canetti* branches [19], showing the prevalence of one of the lineages in Europe and America, the Euro-American lineage, which regroups the strains that had generally been described as principal genetic groups (PGG) 2 and 3 [19].

Since 2004 the genotyping of all clinical isolates of *M. tuberculosis* complex by IS*6110*-based restriction fragment length polymorphism (RFLP) and Spoligotyping in Aragon is systematically performed. Aragon is a region in the Northeast of Spain with 1,345,419 registered inhabitants in the studied year 2010 (http://www.ine.es/jaxi/tabla.do).

The aim of this study was to classify our collection of isolates into SCG lineages, especially those belonging to “U”, “ill-defined” T families and isolates with no family associated. With this intention, we have designed a method based on SNPs detection by multiplex-PCR and pyrosequencing [16,20].

## Methods

### Sample selection

A total of 173 clinical isolates of *M. tuberculosis* complex collected as part of standard patient care from different areas within Aragon in 2010 had been previously identified, susceptibility to first line drugs tested and genotyped by using IS*6110*-RFLP and Spoligotyping techniques. These isolates had been assigned to a lineage or family after have been compared their spoligopatterns with those of the SpolDB4 (fourth international spoligotyping database) [5], in the context of the Surveillance Network monitoring the potential transmission of tuberculosis in Aragon. For the SCG determination assay 101 out of 173 were selected according to the following conditions: only one sample for each RFLP-IS*6110* cluster and the samples with a unique RFLP. Once we confirmed that the isolates with the same spoligopattern were included in the same SCG, a sample selection was made by choosing one isolate for each spoligopattern, resulting in 75 different isolates for further analysis (Table 1). Reference strain H37Rv was included as a control in each test performed.

**Table 1:** Description of the 173 isolates of 2010 in Aragon analysed in this study

**Family based on SpolDB4**		**Isolates genotyped by IS *6110 *-RFLP and spoligotyping (N = 173)**		**Isolates studied by SNPs and classified on SCG (N = 101)**		**Isolates selected based on their different spoligotypes (N = 75)**	
AFRICANUM	AFRI_1	1	1 (0.57%)	1	1 (0.99%)	1	1 (1.33%)
BEIJING	BEIJING	1	1 (0.57%)	1	1 (0.99%)	1	1 (1.33%)
BOVIS	BOVIS1	1	3 (1.7%)	1	3 (2.97%)	1	2 (2.66%)
	BOVIS1_BCG	2		2		1	
CAS	CAS	2	2 (1.25%)	1	1 (0.99%)	1	1 (1.33%)
EAI	EAI7_BGD2	1	1 (0.57%)	1	1 (0.99%)	1	1 (1.33%)
HAARLEM	H1	15	41 (23.6%)	7	25 (24.75%)	6	15 (20%)
	H2	6		2		1	
	H3	19		15		7	
	H3-T3	1		1		1	
LAM	LAM1	1	24 (13.8%)	1	17 (16.83%)	1	10 (13.33%)
	LAM10_CAM	2		1		1	
	LAM12_MAD1	2		1		1	
	LAM2	2		2		1	
	LAM3	5		5		1	
	LAM9	12		7		5	
S	S	4	4 (2.31%)	3	3 (2.97%)	2	2 (2.66%)
X	X1	3	5 (1.15%)	1	2 (1.98%)	1	2 (2.66%)
	X2	2		1		1	
T	T1	27	34 (19.6%)	12	16 (15.84%)	9	13 (17.33%)
	T2	2		1		1	
	T4_CEU1	2		1		1	
	T5	1		1		1	
	T5_MAD2	2		1		1	
U	U	24	26 (15.0%)	10	12 (11.88%)	7	9 (12.00%)
	U (LAM3?)	2		2		2	
No family	NO SIT	31	31 (17.9%)	19	19 (18.81%)	18	18 (24.00%)

The analysis of the DR Region was done in one case in which no positive hybridisation was obtained by spoligotyping using primers DR22-R (5′-AGACGGCACGATTGAGAC) and DR43-F (5′-ACCCGGTGCGATTCTGCG). As no amplification was obtained a deletion of the region in this strain was considered and remains under study. This isolate was considered in the study among the no SIT assigned.

### Analysis of PGGs and SCGs and specific lineage polymorphisms

For the pyrosequencing assay nine SNPs that defined the seven SCGs, were selected from the literature [15]: g.1977A > G, g.74092C > T, g.105139C > A, g.232574G > T, g.311613G > T, g.913274C > G, g.2460626C > A, g.3352929C > G, *and gyrA*95^G→C^ (Table 2). The SNPs presented in *mgtC*^182(CGC→CAC)^*,* in *kat*G^463(CGC→CTG)^ and in *Ag85C*^103(GAG→GAA)^ were identified by sequencing or PCR-RFLP as previously described [8,17,21]. RD^Rio^ deletion was detected by performing a multiplex-PCR [9]. The pattern obtained for the *gyrA*^95^ and *katG*^463^ polymorphisms was coupled to classify each isolate into the different PGGs.

**Table 2:** Base detected at SNPs by pyrosequencing, SCGs and PGGs

**Base at SNP site**										
**1977**	**74092**	**105139**	**232574**	**311613**	**913274**	**2460626**	**3352929**	**gyrA95**	**PGG**	**SCG**
G	C	A	G	T	C	C	G	C	1	2
G	C	C	G	T	C	C	G	C	1	3a
G	C	C	G	T	C	C	G	C	2	3b
G	C	C	T	T	C	C^a^	G^a^	C	2	3c
G	C	C	T	T	C	A^a^	G^a^	C	2	4
G	C	C	G	T	C	C	C	C	2	5
A	C	C	G	T	C	C	C	G	3	6a
A	C	C	G	G	C	C	C	G	3	6b
G	T	C	G	T	G	C	G	C	1	7
G	C	C	G	T	G	C	G	C	1	1
A	C	C	G	T	C	C	G	G	3	6c*

Table adapted from Bouakaze and co-workers [15] and ^a^inferred from Filliol and coworkers [16]. *New pattern SCG-6c.

### Pyrosequencing analysis designed for SNP detection

**Figure 1:**
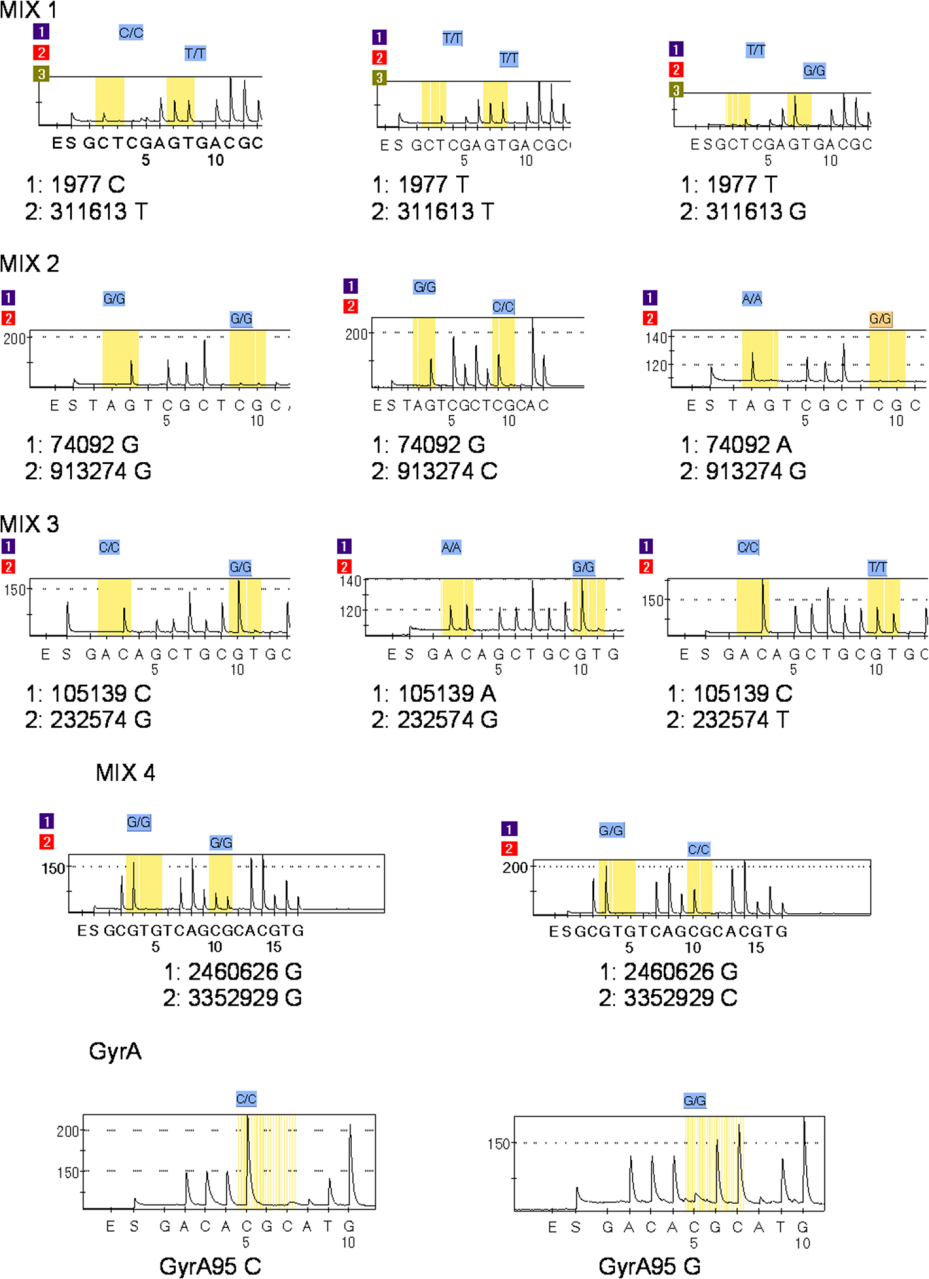
**Pyrograms obtained for different sample assays.** Pyrograms of possible SNP combinations and interpretation for each of the 4 mixed reactions and for the single reactions for detect the *gyrA* polymorphism are shown.

Four multiplex PCR and one simplex PCR were developed to analyse the presence of the nine SNPs within our strains (Figure 1). The SNPs location and gene sequence in H37Rv genome were downloaded from the Tuberculist website (http://tuberculist.epfl.ch/). Primers were designed using the Qiagen® PSQ Assay Design v2.0 software. The programme provided the most suitable primers for DNA amplification, labelling and pyrosequencing, as well as the optimal primer combination in multiplex PCRs (Table 3). For pyrosequencing, an indirect labelling protocol adapted from the literature was followed [20]. First, the PCRs were performed using a universal biotinylated M13 primer and the specific couple of primers (forward and reverse) for each SNP. In a second step, we used the PCR products to pyrosequence them with the subsequent sequencing primer. Each PCR mix contained: 16 mM (NH_4_)_2_SO_4_, 67 mM Tris–HCl pH8.8, 0.01% Tween-20, 1,5 mM MgCl_2_, 200 μM dNTP’, 0.5U SuperHot Taq (Bioron®), 10 pmol of the biotinylated universal M13 primer (5 pmol for GyrA95 PCR mix), 1 μl of each couple of primers (except for 311613-M13:1.3 μl; 232574-M13: 1.5 μl, 913274-M13:1.5 μl) and 1 μl of DNA sample and was adjusted to a final volume of 25 μl with HPLC water. Primers that were not being labelled with biotin in the PCR and the universal M13 primer were used at a concentration of 5 pmol/μl; 25 fmol/μl was used for those having the M13 tail. A 10 pmol/μl concentration was employed for all sequence primers. Amplification was performed in a Veriti® 96-Well Thermal Cycler (Applied Biosystems) for 2 min at 94°C followed by 40 cycles of 15 sec at 94°C, 30 sec at 64°C and 30 sec at 72°C. The amplified products were visualized in a 1.8% agarose gel and were loaded together with a 100 bp molecular weight marker (Bioron*®*). In PCR plates of 96 wells we mixed 40 μl of binding buffer (Qiagen®) and 3 μl of streptavidin-coated Sepharose (*GE-Healthcare®)* beads to the 25 μl of PCR product, and the solution was mixed at 22/23°C for 20–30 min at 1,400 r.p.m. in an *Eppendorf Thermomixer®.* Using the *Vacuum Prep Tool* the biotinylated PCR products were picked up with the 96-filter-unit and consequently immobilized on the streptavidin-coated Sepharose beads. Then, the non-biotinylated DNA was removed by placing the filter unit in the denaturation solution for 5 s, thus generating ssDNA for pyrosequencing. After neutralisation, the vacuum was switched off and the beads containing the PCR product were transferred to a 96-well plate with 16 pmol of each sequencing primer in 40 μl annealing buffer (Qiagen*®*). The sample was transferred into a reaction plate (PSQ 96 Plate Low, Qiagen ®) and incubated for 2 min at 80°C. The volume of enzymes, substrate and nucleotides calculated by *PyroMark Q96 ID* software was added to the PSQ 96 Cartridge accordingly. Pyrosequencing and SNP analysis were done using the PSQ™96MA System and its software (Qiagen®).

**Table 3:** SNP location, primers and PCR designed for pyrosequencing analysis

**PCR primer sequence (5′ → 3′)**					
**Geneª**	**SNP locationª**	**PCR**^**b**^	**Amplicon (bp)**^**b**^	**Forward**^**b**^	**Reverse**^**b**^
*dnaA:dnaN*	1977	Multiplex 1	131	[M13] - TGAGAAGCTCTACGGTTGTTGTTCG	TTTCACCTCACGATGAGTTCGATCC
(Rv0001:Rv0002)					
Rv0260c	311613		114	CACCACTGTTGCCACGATGTTCTT	[M13] - GGCGACTTGCTACGCGTCCTAC
*icd2* (Rv0066c)	74092	Multiplex 2	88	[M13] - GACGGTCCGAATTGCCTTGG	GACCAGGAGAAGGCCATCAAAGAG
*phoT* (Rv0820)	913274		141	GCAATCGCCGTGCAACC	[M13] - CTGCATGTTATGGGTGACGATGAC
Rv0095c	105139	Multiplex 3	94	ATAACGTCGGGCACTGACAAAGAG	[M13]-TCCCGTATCAACTCGTAGGATCTGG
Rv0197	232574		81	CCACGGCGGGGACAAGAT	[M13] -AGAAAGGCGCCGCTGTAGG
*qcrB* (Rv2196)	2460626	Multiplex 4	120	[M13] - GGGCTCGCAGCCAGACTTC	ATGATCACGGCGACCCAGAC
*leuB* (Rv2995c)	3352929		108	[M13] - TCGACGTCCGGGTAGCATTC	GCGTCGCAAGCATCTGACATT
*gyrA* (Rv0006)	codon 95	Simplex	320	CAGCTACATCGACTATGCGA	[M13] - GGGCTTCGGTGTACCTCAT
				**Universal primer**	
				[M13]: CGCCAGGGTTTTCCCAGTCACGAC	

^a^Gene name and SNP location in *M. tuberculosis* H37Rv genome map (http://tuberculist.epfl.ch/). One gene is listed when SNP location is situated in that gene and two genes are listed when SNP is intergenic.

^b^PCR name, amplicon expected size, and primers used.

## Results

We analysed the MTC strain family distribution of 173 isolates collected in 2010 from across Aragon (Table 1). Within this set and according with the spoligotyping analysis, the Haarlem genotype was the most frequent genotype (23.6%), followed by the T *“ill defined”* family (19.6%), U (15%) and LAM (13.8%). Other genotypes showing a defined SIT (9.8%) grouped in smaller groups. Those isolates showing a pattern with no SIT assigned in the spolDB4 database corresponded to 17.9%. Among the 173 isolates, 91 isolates were included in the T, U and no SIT groups representing the 52.6% of the isolates. Accepting those with the same RFLP-IS*6110* genotype as clone-related isolates and therefore belonging to the same family or lineage, only one isolate of each RFLP-IS*6110* genotype, 101 isolates, were analysed by pyrosequencing (Figure 1). Once tested for the presence of the nine SNPs, we could confirm that those isolates with the same spoligopattern held into the same SCG. For further analysis one isolate for each spoligopattern was selected resulting a sample of 75 different MTC strains.

**Figure 2:**
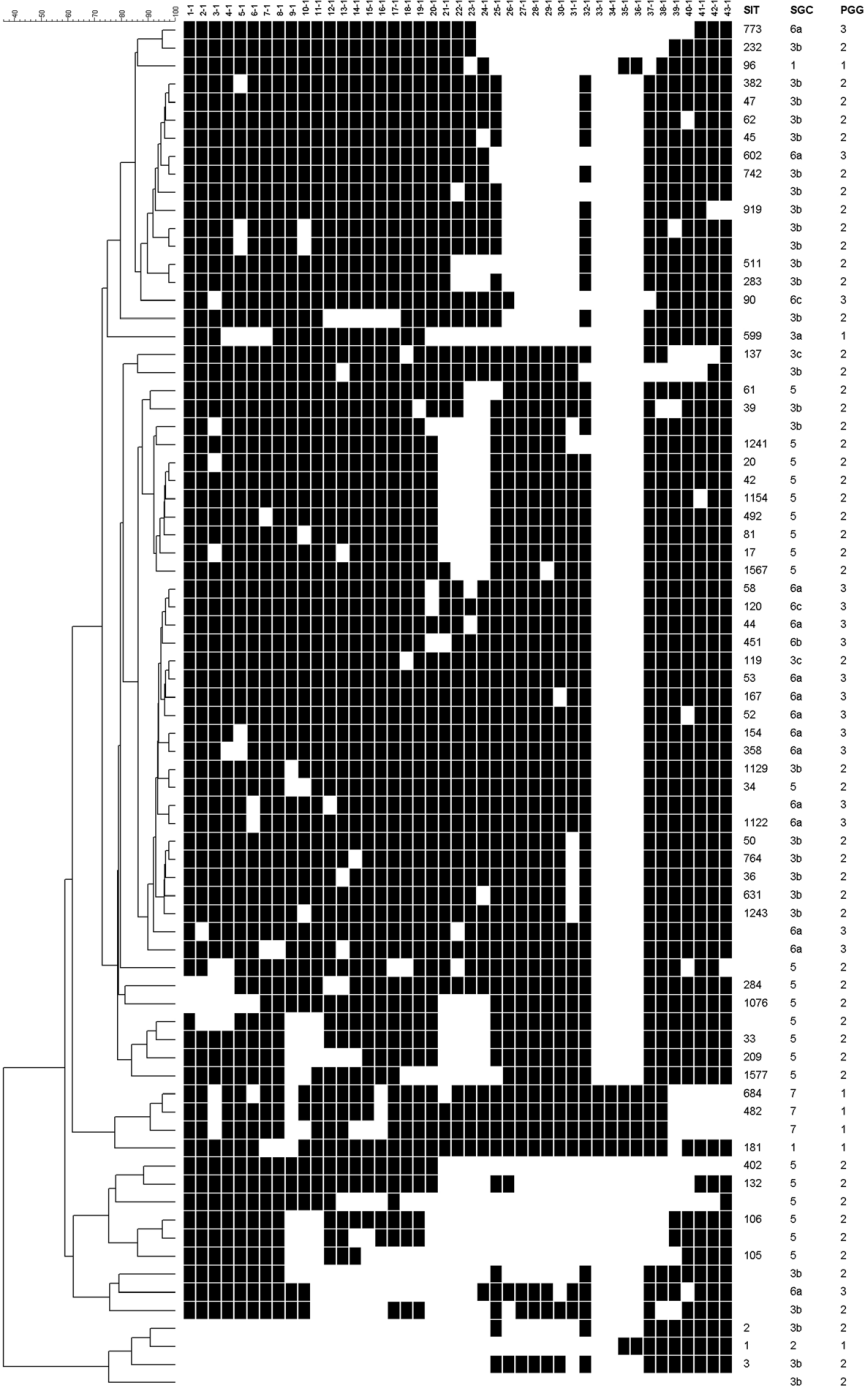
**Dendrogram based on the spoligotypes of the *M. tuberculosis *complex strains studied.** SIT–shared international type, SCG and PGG are detailed. In one isolate a deletion was detected in the DR locus reflected in a negative spoligotype results.

Seven of the 75 strains according with their SNPs in *gyrA* and *katG* genes were found to belong to PGG-1, 52 were included in PGG-2 and 16 were grouped in PGG-3. The strains in PGG-1 shared the SNPs for SCG-7, SCG-1, SCG-2 and SCG-3a. The SCG-3b, SCG-3c and SCG-5 met the feature for PGG-2. Finally, PGG-3 embraced the isolates in SCG-6a and a new SCG that from now on it will be mentioned as “SCG-6c”. The described SCG-6b pattern was only observed for the isolate of H37Rv used as a control. The distribution of these results is drawn and shown in Figure 2 and Table 4. The vast majority of the strains (64 of the 75) were classified in 3 SCGs: SCG-3b, SCG-5 and SCG-6a, in order of relevance. It should be noted that isolates in SCG-4 and SCG-6b were not represented in this study.

**Table 4:** Classification of the 75 clinical isolates analyzed according to PGG and SCG

**SCG**	**1**	**2**	**3a**	**3b**	**3c**	**5**	**6a**	**6b**	6c**	**7**	**Total**
**PGG 1**	2	1	1							3	7
**PGG 2**				27	2	23					52
**PGG 3**							14	*	2		16
											**75**

*Reference strain H37Rv. **New SCG subgroup reported.

**Figure 3:**
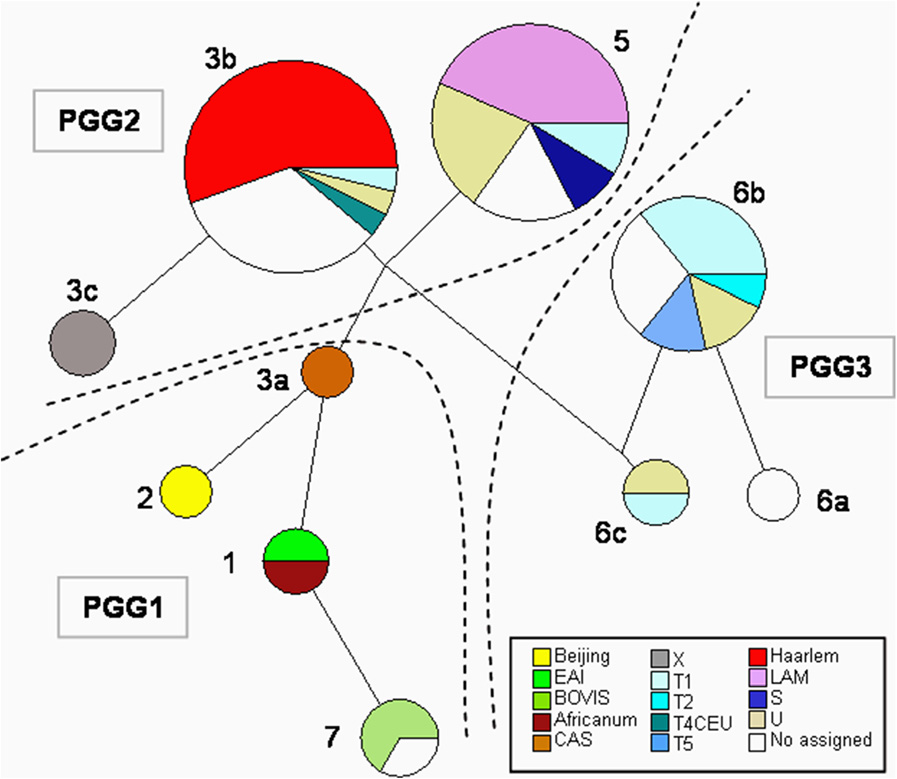
**Phylogenetic tree based on the 9 SNPs selected for SCGs.** Model-based neighbour-joining tree based on the 9 SNPs resolved of the 75 *M. tuberculosis* complex isolates and the reference strain analysed into the different SCGs. Numbers designate each SCG and Spoligotyping families are indicated by a different colour detailed in the legend. The SNP lineages that belong to the three “major genetic” groups based on combination of two alleles at *katG463* and *gyrA95* are also highlighted. The scale bar indicates the number of SNP difference.

Regarding the spoligo-families detected (Figure 3), the unique isolates in our study belonging to AFRI_1 and EAI7_BGD2 families were grouped in SCG-1. The Beijing strain corresponded to the SCG-2 and the unique CAS isolate was included in SCG-3a. The *M. bovis*-BCG and *M. bovis* isolates (for one of them the SIT was not assigned) were grouped into SCG-7. The fifteen cases known to belong to the Haarlem family were grouped in SCG-3b. The 10 LAM and also the two S family strains were classified in SCG-5. Two cases belonging to the X family were included in SCG-3c. Our results showed that the 40 strains previously classified by Spoligotyping in the *ill-defined* T, U family or with no SIT assigned, were distributed among SCG-3b, SCG-7, SCG-5, SCG6-a and SCG-6c (Table 5).

**Table 5:** Phylogenetic distribution of the T, U and with no SIT isolates according to their SCG

**SCG**	**Family**	**T**					**U**		**No SIT**	**Total**
		**T1**	**T2**	**T4-CEU1**	**T5**	**T5-MAD2**	**U**	**U (LAM3)**		
**3b**	Haarlem						1		7	8
	No Haarlem	1		1					2	4
**7**	BOVIS								1	1
**5**	LAM	1					3	2	3	9
	No LAM	1							1	2
**6a**	“Authentic” T	5	1		1	1	2		4	14
**6c**	New pattern	1					1			2
**Total**		**9**	**1**	**1**	**1**	**1**	**7**	**2**	**18**	**40**

SCG-3b included twelve isolates, nine of them were not assigned to any of the spoligo-families, one isolate belonged to T1 family (SIT 1129), one isolate to T4_CEU1 family (SIT 39) and one isolate to U family (SIT 232). Furthermore, additional SNP at codon 182 in *mgtC* gene specific to the Haarlem family was studied in these strains. The codon *mgtC*^*182*(CAG)^ was present in eight of these isolates, including the classified as SIT 232.

SCG-5 included eleven isolates of T1 (SIT 284 and 1567), U (SIT 132, 402 and 1241) and U-LAM3 (SIT 105 and 106) families and four isolates which did not have any SIT assigned. They were studied to settle on their LAM family membership. All of them except two (SIT 284 and other with no SIT assigned) presented the LAM specific SNP in *Ag85*C^103(GAG→GAA)^. In addition, we found that two among the isolates tested, or five considering all the LAM strains, contained the RD^Rio^ deletion, which is a feature of a subgroup of the LAM family strains.

SCG-6a included a total of 14 isolates, which belonged to T1 (SIT 53, 154, 167, 358, 1122), T2 (SIT 52), T5 (SIT 44), T5_MAD2 (SIT 58), U (SIT 602 and 773) and 4 isolates with not SIT assigned. None of them had either the SNP in *Ag85*C^103^ or the SNP in *mgtC*^*182*^. This SCG-6a included the isolate of the most representative cluster in 2010, ARA7 (SIT 773, U family), which gathered 133 clinical cases since 2004 [22]. Finally, two unrelated and different isolates presented the same new pattern named SCG-6c, which only differs from SCG-6a in one SNP (Table 2). The first isolate (SIT 90, U) was related with the outbreak ARA21 (20 cases collected since 2004) and the second isolate (SIT 120, T1 family) had not been previously reported in our Region. Neither contained the SNP in Ag85C^103^ nor the SNP in *mgtC*^182^ feature for LAM or Haarlem families respectively.

## Discussion

The Euro-American lineage was found to be the predominant lineage of the *M. tuberculosis* complex in Europe [19]. The MDR TB studies carried out in Spain showed the Euro-American as the more prevalent lineage [23], and that a few LAM and Haarlem strains, which belong to this lineage, played a major role in the spread of MDR strains [24]. According to this, the 90% of the tuberculosis strains analysed in this work belong to this lineage. Our work allowed to classify a collection of MTC strains previously analysed by Spoligotyping and RFLP in Aragon in lineages as well as in SCGs by the detection of the 9 SNPs that define the 7 SCGs [15,16] together with PCR identification of katG^463^, Ag85C^103^ and mgtC^182^ polymorphisms. All these single polymorphisms as a whole have proved to be an effective complement for both Spoligotyping and RFLP techniques that enhance their sensibility, especially in those families identified at the beginning as T, U and orphan. A notorious circumstance to remark in our population was that the two largest clusters of *M. tuberculosis* strains, named ARA21 and ARA7, belonged to T and unclassified groups of families. Besides, ARA7 had caused an outbreak since 2004, what resulted in around the 20% of cases of tuberculosis [22]. This fact allows the classification of these strains into more resolved families. In addition, the 9 SNPs detection by using a pyrosequencing assay leads to obtain quick and reliable results at an affordable cost [20].

We have shown that some strains identified by Spoligotyping as T, U or even orphan, which represent in our study the 52.6% of the isolates, belong in fact to defined families that could be assigned by using the aforementioned polymorphism set. In few occasions it was not possible to group those strains into a family with certainty, therefore SNP detection in Ag85C^103^ and mgtC^182^ was needed. Thus, regarding SCG-3b, the most prevalent in our community, the addition of a specific SNP detection as mgtC^182^, a characteristic SNP of the Haarlem family, gave more specific information. Filliol and collaborators joined in this SCG-3b basically Haarlem isolates, but also some T, LAM, and orphan strains [16]. It either happened the same concerning SCG-5, the second most prevalent SCG in Aragon, in which Filliol and collaborators included essentially LAM strains, but also T, Haarlem, S, unknown and orphan isolates [16]. The pyrosequencing method applied allows to include an isolate in SCG-5, further the *Ag85C*^103^ asserts of its LAM membership even if spoligotyping had not been detected it at first. Regarding SCG-6a, which was the third group of relevance in our study, we believe it includes the vast majority of the T isolates that would group as the “authentic T” isolates, being a more evolved strains since they belong to the PGG-3. Another achievement of this SNPs set has been the discovery of the two genetically and epidemiologically not linked isolates included in the new “SCG-6c”. It suggests that the tubercle bacillus is incessantly varying and highlights the value of SNPs to follow the evolution of *M. tuberculosis* complex.

Concerning the PGG determination, around 70% of the strains circulating in our community grouped in the PGG-2. This study provides a first inside into the structure of the *M. tuberculosis* population in Aragon and Spain. The strains causing the largest clusters were classified as belonged to PGG-3, ARA7 (SCG-6a) and ARA21 (SCG-6c), what means these modern strains are causing the more cases of TB in our region, both of them belong to the Euro-American lineage [19,25]. Comparing our results with a study carried out in London [26], we appreciate less diversity regarding Spoligo-families probably due to the minor rate of patients that born abroad in respect to the London population. They characterised the MTBC strains using SNPs, however some of the isolates remained unclassified. A recent publication designed an algorithmic differentiating Euro-American based on polymorphic SNPs in 5 genes in an extend collection of well-classified members of the MTB complex [27]. However, the application of the analysis of the set of SNPs previously described [8,17,21] selected in this study allowed us to assign 75 strains sharing different spoligotypes to different SCGs and families in the MTC, specially those assigned to the ill defined T and other unclassified. We believe that classifying our isolates in the precedent PGGs previously described along with the SCGs and spoligo-families provided the appropriate information to better understand the phylogenetic background of the Aragonian strains being this approach applicable to other isolates of any geographical location.

## Conclusions

In conclusion, the current study shows that the polymorphisms selected have been quite useful to complement and enrich the characterization of all isolates, specifically for those that would not have been classified by other routine techniques. Although more studies with a larger amount of samples would be required, this work has allowed us to do a better classification of Aragonian strains into SCGs and PGGs by using pyrosequencing and conventional PCR, and in some cases, to assign strains to a certain lineage. Besides, the description of a new pattern shared by two isolates “SCG-6c” reinforces the interest of SNPs to follow the evolution of *M. tuberculosis* complex. In addition, our work describes the successful development of a multiplex-PCR and pyrosequencing assay based on SNP detection as a purpose to classify *M. tuberculosis* isolates into more resolved phylogenetic groups called SCGs and to determine the principal genetic groups. Therefore we suggest the use of this pyrosequencing technique as a complement to current phylogenetic and epidemiological investigations.

## Ethics statement

The Ethical Committee of the Aragon Government approved the study and the protocols for collecting the bacterial strains from patients. Any human sample was collected.

## Abbreviations

MTC: Mycobacterium tuberculosis complex; LSPs: Long sequence polymorphisms; SNPs: Single nucleotide polymorphisms; PGG: Principal genetic group; DR: Direct repeats; LAM: Latin American-Mediterranean family; TB: Tuberculosis; SIT: Shared international type; MDR: Multidrug resistant; CAS: Central Asian family; EAI: East African Indonesian family; SCG: SNP cluster groups; RFLP: Restriction fragment length polymorphism; SpolDB4: Fourth international *spoligotyping* database.

## Competing interests

None of the investigators has any financial interest or financial conflict with the subject matter or materials discussed in this report. All authors read and approved the final manuscript.

## Authors’ contributions

SS and JD contributed to the study design, AC, MS design and the development of the pyrosequencing technique, CM, MJI, MAL, MAV, MF facilitate the background and support the mycobacterial isolates genotyping studies. AC and SS analysed data and drafted the manuscript. All authors read and approved the final manuscript.
